# Novel Molecular Evidence Related to COVID-19 in Patients with Diabetes Mellitus

**DOI:** 10.3390/jcm9123962

**Published:** 2020-12-07

**Authors:** Yu-Huang Liao, Jing-Quan Zheng, Cai-Mei Zheng, Kuo-Cheng Lu, You-Chen Chao

**Affiliations:** 1 Division of Endocrinology and Metabolism, Department of Internal Medicine, Taipei Tzu Chi Hospital, Buddhist Tzu Chi Medical Foundation, New Taipei City 231, Taiwan; lyuh5398@gmail.com; 2 Division of Pulmonary Medicine, Department of Internal Medicine, Shuang Ho Hospital, Taipei Medical University, New Taipei City 23561, Taiwan; 16044@s.tmu.edu.tw; 3 Division of Pulmonary Medicine, Department of Internal Medicine, School of Medicine, College of Medicine, Taipei Medical University, Taipei 11031, Taiwan; 4 Graduate Institute of Clinical Medicine, College of Medicine, Taipei Medical University, Taipei 110, Taiwan; 5 Research Center of Urology and Kidney, Taipei Medical University, Taipei 110, Taiwan; 11044@s.tmu.edu.tw; 6 Division of Nephrology, Department of Internal Medicine, Taipei Medical University Shuang Ho Hospital, New Taipei City 235, Taiwan; 7 Division of Nephrology, Department of Internal Medicine, School of Medicine, College of Medicine, Taipei Medical University, Taipei 110, Taiwan; 8 Division of Nephrology, Department of Internal Medicine, Taipei Tzu Chi Hospital, Buddhist Tzu Chi Medical Foundation, New Taipei City 231, Taiwan; kuochenglu@gmail.com; 9 School of Medicine, Tzu Chi University, Hualien 970, Taiwan; 10 Division of Gastroenterology, Department of Internal Medicine, Taipei Tzu Chi Hospital, Buddhist Tzu Chi Medical Foundation, New Taipei City 231, Taiwan

**Keywords:** coronavirus disease 2019, severe acute respiratory syndrome coronavirus 2, diabetes mellitus, hyperglycemia, Innate immunity, angiotensin-converting enzyme 2, renin–angiotensin–aldosterone system

## Abstract

Coronavirus disease 2019 (COVID-19), caused by severe acute respiratory syndrome coronavirus 2 (SARS-CoV-2), has rapidly evolved into a global pandemic. The hyperglycemia in patients with diabetes mellitus (DM) substantially compromises their innate immune system. SARS-CoV-2 uses human angiotensin-converting enzyme 2 (ACE2) receptors to enter the affected cell. Uncontrolled hyperglycemia-induced glycosylation of ACE2 and the S protein of SARS-CoV-2 could facilitate the binding of S protein to ACE2, enabling viral entry. Downregulation of ACE2 activity secondary to SARS-CoV-2 infection, with consequent accumulation of angiotensin II and metabolites, eventually leads to poor outcomes. The altered binding of ACE2 with SARS-CoV-2 and the compromised innate immunity of patients with DM increase their susceptibility to COVID-19; COVID-19 induces pancreatic β-cell injury and poor glycemic control, which further compromises the immune response and aggravates hyperglycemia and COVID-19 progression, forming a vicious cycle. Sequential cleavage of viral S protein by furin and transmembrane serine protease 2 (TMPRSS2) triggers viral entry to release the viral genome into the target cell. Hence, TMPRSS2 and furin are possible drug targets. As type 1 DM exhibits a Th1-driven autoimmune process, the relatively lower mortality of COVID-19 in type 1 DM compared to type 2 DM might be attributed to an imbalance between Th1 and Th2 immunity. The anti-inflammatory effects of dipeptidyl peptidase-4 inhibitor may benefit patients with DM and COVID-19. The potential protective effects of sodium–glucose cotransporter-2 inhibitor (SGLT2i), including reduction in lactate level, prevention of lowering of cytosolic pH and reduction in pro-inflammatory cytokine levels may justify the provision of SGLT2i to patients with DM and mild or asymptomatic COVID-19. For patients with DM and COVID-19 who require hospitalization, insulin-based treatment is recommended with cessation of metformin and SGLT2i. Further evidence from randomized or case–control clinical trials is necessary to elucidate the effectiveness and pitfalls of different types of medication for DM.

## 1. Introduction

Coronavirus disease 2019 (COVID-19), an infection caused by an enveloped virus with a single-stranded RNA genome, namely novel severe acute respiratory syndrome coronavirus 2 (SARS-CoV-2), has caused a global pandemic. Several physical conditions, including older age, severe obesity, and diabetes mellitus (DM) are associated with an increased risk of morbidity and death from COVID-19 [1]. Individuals with DM have a higher risk of nosocomial bacteremia, pulmonary infection and infectious disease because of reduced immune response. The impairments of the innate and humoral immune systems secondary to metabolic dysfunction in patients with DM render them susceptible to infectious diseases [2]. As patients with DM were associated with a poor prognosis of COVID-19 and COVID-19 tends to worsen the dysglycemia of these patients, complex interactions between DM and COVID-19 were proposed [3]. Researchers have sought to elucidate the association between COVID-19 and DM in order to improve the understanding of the potential mechanism and provide effective treatment for patients with DM and COVID-19. In this review, we discuss the possible pathophysiology and mechanisms, clinical characteristics and potential concerns regarding treatment choices for patients with DM and COVID-19.

## 2. Crosslink Between Hyperglycemia/DM and COVID-19

**Figure 1:**
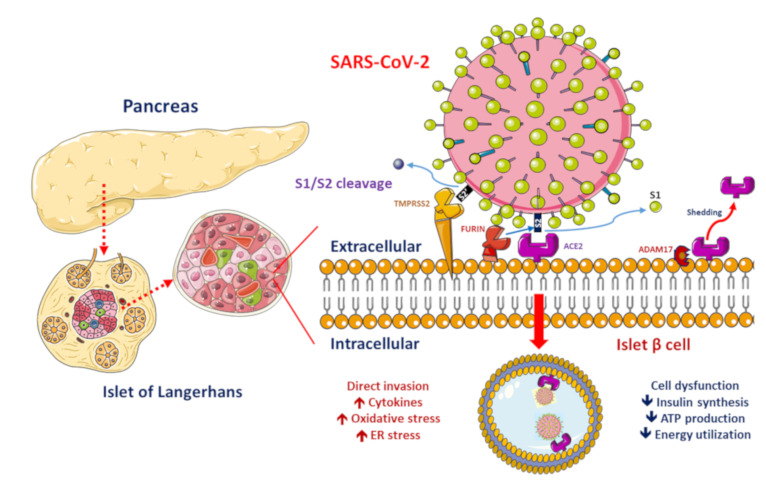
Proposed functions of pancreatic β-cell molecules for interaction with severe acute respiratory syndrome coronavirus 2 (SARS-CoV-2).

Patients with DM have a risk of developing more severe COVID-19 [4]. In addition, patients with COVID-19 experience abnormalities of glucose metabolism, such as hyperglycemia, euglycemic ketosis and even classic diabetic ketoacidosis [5]. There are complicated crosslinks between hyperglycemia/DM and COVID-19 (Figure 1). Patients with DM have an increased risk of requiring critical care in the intensive care unit, requiring invasive mechanical ventilation, or mortality after infection with SARS-CoV-2 [6]. Hyperglycemia in these patients can significantly compromise their innate immune responses to infection [7]. Among patients with COVID-19, compared with those without DM, those with DM had higher levels of inflammation markers, including interleukin-6, C-reactive protein and ferritin, which suggested that the pro-inflammatory state of patients with DM was prone to rapid deterioration and subsequently poor COVID-19 outcomes [8].

ACE2, ADAM17 and TMPRSS2/FURIN are present in the β-cell membrane in type 2 diabetic human pancreatic islets. The SARS-CoV-2 spike protein should be cleaved at two sites, namely S1/S2 and S2′, to trigger the fusion of viral and cellular membranes during virus entry to release the virus genome into the host cell. Initially, cleavage at the S1/S2 site by TMPRSS2 occurs, followed by cleavage at the S2′ site by FURIN. SARS-CoV-2 replication could be inhibited by the synthetic FURIN inhibitor. The direct binding of SARS-CoV-2 to ACE2 on β-cells might contribute to β-cell damage and insulin deficiency, which, combined with cytokine-induced insulin resistance and hypokalemia-related inhibition of insulin secretion, contributes to worsening glucose control in patients with diabetes mellitus.

**Figure 2:**
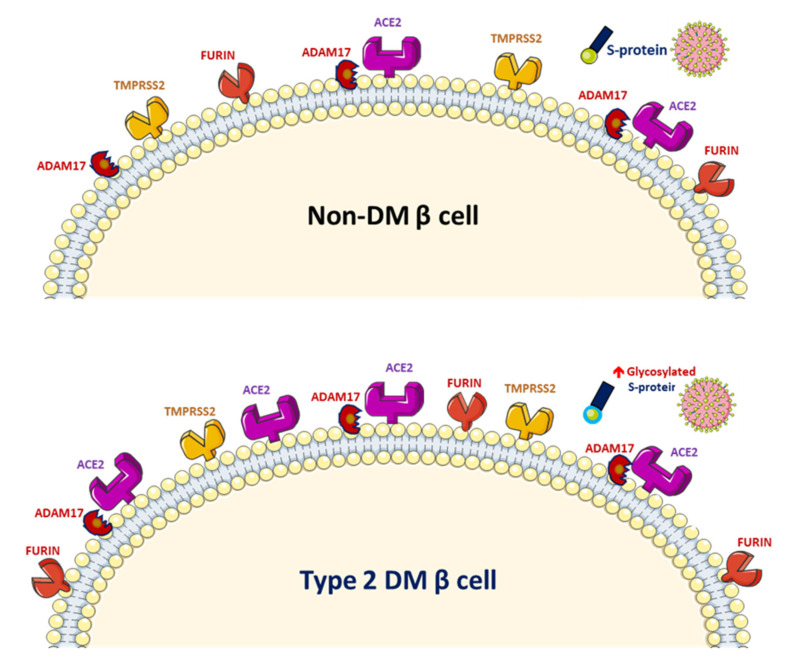
Increased glycosylated ACE2, ADAM17 and TMPRSS2 expression in human pancreatic islets and glycated SARS-CoV-2 spike protein in patients with diabetes mellitus (DM).

SARS-CoV-2 uses the human angiotensin-converting enzyme 2 (ACE2) receptors to enter the affected cell [9]. ACE2 exists in the human body in both membrane-bound and soluble forms and viral entry of SARS-CoV-2 into host cells relies on binding of the viral spike (S) proteins to the membrane of ACE2 [10]. A previous study has reported increased activity of ACE2 in diabetic mice [11]. A phenome-wide Mendelian randomization study reported a causal link between DM and the increased expression of ACE2 [12]. Increased ACE2 levels were also found in patients with cardiovascular disease or those taking renin–angiotensin system (RAS) blockers [13]. A marked increase in mortality among DM patient with COVID-19 was demonstrated in a recent meta-analysis [14]. On the other hand, obesity and metabolic disorders play an important role in COVID-19 prognosis since the larger amount of ACE2 receptors in adipocytes serve as a reservoir for the SARS-CoV-2 virus [15,16]. However, it is still unclear whether solely elevated ACE2 levels pose a higher risk of SARS-CoV-2 infection. The downregulation of ACE2 activity secondary to SARS-CoV-2 infection with consequent accumulation of angiotensin II and metabolites might eventually lead to acute respiratory distress syndrome or fulminant myocarditis [13]. ACE2 converts angiotensin II to angiotensin 1–7 (Ang 1–7), which promotes vasodilation, and enhanced ACE2 activity has been shown to mediate a protective effect in patients with COVID-19 based on anti-inflammatory benefits due to the enhancement of ACE2 [17]. Therefore, the amount of glycosylated ACE2 receptor rather than total ACE2 receptor in patients with DM is likely related to the augmented viral entry [18]. Previous studies have shown a strong link between hyperglycemia and the severity of COVID-19 [19] and severe acute respiratory syndrome (SARS) [20]. A recent study demonstrated that glycosylation of the ACE2 receptor in humans substantially contributes to the binding of the SARS-CoV-2 (Figure 2) [21]. Structure–function studies have also shown that the S protein of SARS-CoV-2 is highly glycosylated [22]. Uncontrolled hyperglycemia might induce potential modifications in glycosylation of ACE2 and the S protein of SARS-CoV-2, which possibly enables the S protein to bind to ACE2 and alters the immune response to the virus [18]. A recent study proposed that the worse outcomes in patients with DM who develop COVID-19 could be related to the nonenzymatic glycation of ACE2 [23]. Therefore, patients with high blood glucose or DM are at a risk of developing more severe COVID-19, which might be attributed to the hyperglycemia related aberrant glycosylation or hyperglycemia-enhanced nonenzymatic glycation of ACE2 and SARS-CoV-2 spike protein.

Both RAS and ACE2 play important roles in DM patients with COVID-19, especially through bradykinin being degraded by ACE and angiotensin 1–9 being activated by ACE2. Further, an atypical pattern of the RAS in COVID-19 patients was noted with a decreased ACE/ACE2 ratio and increased renin, angiotensin, kininogen, bradykinin and bradykinin receptors, which altogether induced hypotension, vasodilation, etc. and most of the symptoms related to patients with COVID-19 [24].

Worsening insulin resistance and glycemic control have been observed in patients with DM who develop SARS-CoV-2 infection, likely owing to the pro-inflammatory state induced by COVID-19 [3]. The elevated cytokine levels secondary to SARS-CoV-2 infection may cause impairments in pancreatic β-cell function and apoptosis [25]. In addition, ACE2 expression in the pancreas has been reported [26]. SARS-CoV was detected in the pancreas of patients whose death was attributed to SARS [27]. These findings suggest that direct binding of SARS-CoV-2 to ACE2 on pancreatic β-cells might contribute to their damage (Figure 1) and subsequent insulin deficiency and hyperglycemia, as observed previously in patients with SARS-CoV infection [28]. Available evidence shows that the direct β-cell damage [28], inflammation-induced insulin resistance [29], hypokalemia-related inhibition of insulin secretion by β-cells [30] and corticosteroid use in the treatment of COVID-19 contribute to the poor glycemic control in patients with DM who develop COVID-19 [3]. A previous study reported that patients with DM who were on insulin therapy required higher doses of insulin and those who were on oral antidiabetics required insulin therapy after admission owing to COVID-19 [8]. Hence, a vicious cycle secondary to SARS-CoV-2 infection in patients with DM was proposed, in that the altered ACE2 binding of SARS-CoV-2 and compromised innate immunity of patients with DM increase their susceptibility to COVID-19 and COVID-19 induces pancreatic β-cell injury leading to a worse glycemic profile, which, in turn, impairs the immune response, induces a proinflammatory state and eventually aggravates COVID-19 progression and poor glycemic control.

Several molecular mechanisms regarding the correlations between glucose metabolism and SARS-CoV-2 replication have been proposed. After viral entry of SARS-CoV-2 into human cells, glucose availability affects viral replication [31] and the utilization of excess glucose facilitates viral replication through the hexosamine biosynthetic pathway (HBP), which again induces overexpression of interferon regulatory factor–5 (IRF5), leading to subsequent endoplasmic reticulum stress, overproduction of cytokines, hyperinflammation and eventually multiorgan failure [32]. Among patients infected with influenza A virus, higher proinflammatory cytokines significantly correlated with increased sugar levels due to the essential function of HBP [33]. Evident findings revealed that IRF5 expression could be induced by persistent hyperglycemia, repetitive intermittent hyperglycemia and glucose fluctuations [34] and the contribution of daily postprandial glucose peaks after meals to the SARS-CoV-2 replication was proposed. These molecular mechanisms might provide hints for potential therapeutic strategies.

## 3. Clinical Characteristics and Outcomes of Patients with DM who Develop COVID-19

Compared with hospitalized patients with COVID-19 without DM, those with DM have worse clinical profiles and outcomes, such as higher blood glucose, HbA1c, white blood cell count, high-sensitivity C-reactive protein, procalcitonin, ferritin, D-dimer, lactic dehydrogenase, N-terminal pro-brain natriuretic peptide and mortality [35]. These findings indicate that patients with COVID-19 with underlying DM had worse glycemic control, more severe infection and myocardial damage. The prevalence of DM in patients with mild COVID-19 was estimated to be 5.7%, which increased to 16.2% in those with severe COVID-19 [36]. Compared with patients with COVID-19 who did not have DM, those with underlying DM had a higher risk of requiring critical care in the intensive care unit, invasive mechanical ventilation and mortality [6].

### 3.1. Diabetes, Respiratory Infections and COVID-19

Over the past few decades, DM has been shown to be a risk factor for mortality in patients with H1N1 influenza, severe acute respiratory syndrome (SARS) and Middle East respiratory syndrome (MERS) [37]. In an animal model study of MERS, diabetic mice had dysregulated immune responses and fewer monocytes, macrophages and CD4^+^ T cells [38]. Patients with severe COVID-19 and underlying DM had lower CD4^+^ T lymphocyte counts than did those with non-severe COVID-19 [39]. Other than the compromised immune response in patients with DM, enhanced viral entry and reduced viral clearance could account for their increased susceptibility to COVID-19. Furin, a member of the subtilisin-like proprotein convertase family, is a type 1 membrane-bound protease present in several organs [40]. After viral S protein binds to ACE2, furin cleaves the S protein at a cleavage site between the S1/S2 subunits, a process that is required for viral entry (Figure 1) [22]. Thus, inhibition of furin expression could be a potential strategy to prevent COVID-19. However, a previous study showed that furin levels are increased in DM [40], which may contribute to the high risk of the SARS-CoV-2 infection in patients with DM. In a recent study, DM was found to adversely affect SARS-CoV-2 viral clearance [41]. In summary, altered immune response, augmented viral entry and decreased viral clearance in patients with DM contribute to their higher susceptibility to COVID-19.

#### 3.1.1. Role of TMPRSS2 and Furin in Proteolytic Activation of SARS-CoV-2

When SARS-CoV-2 enters cells, S glycoprotein must be cleaved by proteases at two different sites, namely S1/S2 and S2′ [42]. A previous in vitro study proposed that several proteases participate in the activation of CoVs, such as furin, cathepsin L, transmembrane serine protease 2 (TMPRSS2), TMPRSS11a and TMPRSS11d [43]. The cleavage of the spike protein by furin or TMPRSS2 initiates the ACE2-dependent viral entry at the cell membrane. During the intracellular phase, the activation of S protein is triggered by cathepsin in lysosomes or furin in the trans-Golgi network [44]. A recent study demonstrated that when SARS-CoV-2 enters cells, the sequential cleavage of viral S protein at the S1/S2 site by furin and the S2′ site by TMPRSS2 triggers viral entry to release the viral genome into the target cell. This suggests that TMPRSS2 and furin are possible therapeutic targets. A potential TMPRSS2 inhibitor, nafamostat mesylate, inhibited TMPRSS2-dependent cell entry of MERS-CoV [45] and potently inhibited SARS-CoV-2 S protein-mediated fusion in vitro [46] and the synthetic furin inhibitor MI-1851 shows promise in inhibiting SARS-CoV-2 replication in human respiratory tract cells [42].

#### 3.1.2. COVID-19 and Pancreatic Islet β-Cells in Type 2 DM

Activated local RAS in DM causes enhanced oxidative stress injury to pancreatic β-cells. The ACE2/Ang (1–7)/Mas axis mediates negative regulation of the classical RAS [47]. Pancreatic ACE2 plays an essential role in maintaining glycemia and β-cell function. Induced expression of ACE2 in pancreatic islet β-cells clearly demonstrated beneficial effects in treating DM [48]. In obese C57BL/6 mice models, ACE2 deficiency reduced β-cell mass and impaired β-cell proliferation. However, ACE2 overexpression in the pancreas of diabetic mice improved glycemic control [49]. In a high-fat-diet-induced mouse model, enhancement of the ACE2/Ang 1–7 axis attenuated β-cell dedifferentiation, which protects against metabolic stress; the protection might be partially explained by improvements in islet microcirculation and suppression of islet-inducible nitric oxide synthase [50]. Another cellular study revealed that islets from Ace2^-/y^ mice showed impaired mitochondrial respiration with lower production of ATP and reduced expression of genes involving mitochondrial oxidation. However, ACE2 overexpression observed in islets from db/db mice restored impaired mitochondrial oxidation and potentially increased glucose-stimulated insulin secretion [51]. A study on pancreatic β-cells (INS-1 insulinoma cells) also reported that Ang 1–7 mediated the protective effects on pancreatic β-cells through its antioxidant effects [47].

ACE2 shedding from the cell membrane could be mediated by disintegrin and metalloproteinase 17 (ADAM17) (Figure 1). ADAM17 overexpression leads to increased shedding of the membrane form of ACE2 and reduced cellular ACE2 levels in mouse pancreatic islet cells. ADAM17 causes cleavage of ACE2 only when both are co-expressed in the same cell. In all types of pancreatic islet cells, the β-cell is not the major cell type expressing ACE2 and both ADAM17 and ACE2 are expressed in pancreatic islets [52]. In another study, the expression pattern of ACE2, ADAM17 and TMPRSS2 in type 2 diabetic human pancreatic islets derived from recent microarray and RNA-sequencing expression data showed that pancreatic islets expressed all three receptors and higher ACE2 expression was found in sorted pancreatic β-cells, compared with that of other endocrine cells. Compared with nondiabetic/normoglycemic cells, diabetic/hyperglycemic islets showed significantly increased expression of ACE2. Thus, ACE2 expression is augmented in the islets of people with diabetes. However, whether variations of ACE2 expression could explain the severity of SARS-CoV-2-infection-related symptoms warrants further elucidation [53]. Recent evidence regarding the expression of ACE2 and TMPRSS2 in the human pancreas showed intriguing findings that the ACE2 expression was found in islet and exocrine tissue microvasculature and in a subset of pancreatic ducts, whereas TMPRSS2 expression was limited to ductal cells. The researchers found that neither protein was detected in β-cells, which argues against direct SARS-CoV-2 viral infection to β-cells in vivo via ACE2 and TMPRSS2 [54]. Since there is ambiguous evidence regarding the expression and role of ACE2, ADAM17 and TMPRSS2 in the human pancreas during the course of SARS-CoV-2-infection, further studies are warranted.

#### 3.1.3. Type 1 DM May be Less Lethal than Type 2 DM in COVID-19 Infection

While COVID-19 is rapidly spreading worldwide, a vulnerable population of patients with type 1 DM seem to have a lower mortality than those with type 2 DM in the course of COVID-19. Preliminary data focused on type 1 DM patients with COVID-19 demonstrated that nearly one-third of patients suffered from diabetic ketoacidosis (DKA) and the mortality rate was 3.2% [55], which seems to be relatively lower than the rate (10%) in patients with type 2 DM [6]. Type 1 DM exhibits a Th1-driven autoimmune process, as observed in a patient with Hashimoto’s thyroiditis, which frequently coexists with type 1 DM. In the pathogenesis of type 1 DM, the destruction of β-cells is mediated by islet-specific CD8^+^ cytotoxic T cells in a cytokine microenvironment provided by diabetogenic CD4^+^ Th1 [56]. Thus, the less lethal presentation of COVID-19 in type 1 DM compared to type 2 DM might be attributed to an imbalance between Th1 and Th2 immunity [57].

### 3.2. Special Considerations for Patients with DM during the COVID-19 Pandemic

**Figure 3:**
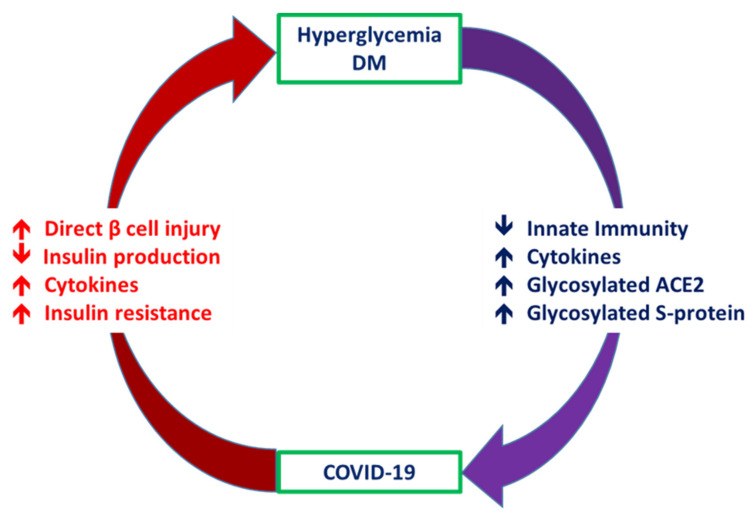
Crosslink between Coronavirus disease 2019 (COVID-19) and diabetes mellitus.

Owing to the complex correlation between DM and COVID-19, glycemic management plays an important role in the clinical course of COVID-19. In a previous study on SARS, DM and hyperglycemia were independent predictors of morbidity and mortality in patients with SARS [20]. Another study reported that the SARS coronavirus uses ACE2 as its receptor to enter islet cells and damage them [28]. One study reported that replication of influenza virus increased with higher blood glucose levels in diabetic mice and the augmented susceptibility was reduced by insulin [58]. As a vicious cycle occurs between SARS-CoV-2 infection and DM, a comprehensive management program to enhance glycemic control in patients with DM who develop COVID-19 is warranted (Figure 3).

Hyperglycemia/diabetes mellitus contributes to increased disease severity of COVID-19 via reduction in innate immunity, magnified proinflammatory cytokine response and high expression of glycated ACE2. Therefore, diabetes mellitus and hyperglycemia have widely been recognized to aggravate the severity of COVID-19. COVID-19 leads to deterioration of glucose control in patients with diabetes mellitus, likely through direct virus-mediated β-cell injury, cytokine-induced insulin resistance and hypokalemia-related inhibition of insulin secretion.

#### 3.2.1. Changes to DM Management amid the COVID-19 Pandemic

Owing to the lack of curative therapy for COVID-19 at present, patients with DM should closely monitor their own glycemic profile and take extra precautions to maintain glycemic control. Education regarding hand hygiene and social distancing should be universally provided. As the widespread lockdown measures may alter the physical activity, diet and clinic visits of patients with DM, extra remote measures to provide regular medical service and professional consultation should be adopted. Considering the adverse effect on the mental health of patients with DM during the COVID-19 pandemic, a timely psychological intervention that would benefit patients in need should be introduced.

#### 3.2.2. Management of Diabetic Ketoacidosis during COVID-19

An unusually high number of patients with COVID-19 were reported to have diabetic ketoacidosis (DKA) or a hyperosmolar hyperglycemic state during the early stages of the COVID-19 pandemic [59]. It was proposed that DKA was caused by β-cell-damage-induced insulin deficiency caused by the SARS-CoV-2 infection [60]. Subcutaneous insulin therapy was suggested for patients with COVID-19 who had DKA and in whom intravenous insulin infusion was not possible [59]. As lactic acidosis associated with metformin and DKA associated with SGLT-2 inhibitors may pose a risk of metabolic acidosis development, these drugs should be discontinued in patients with DM who have severe COVID-19. Experiments on SARS-CoV-infected mice showed prominent lung microvascular leakage after infection [61]. Therefore, the traditional aggressive fluid replacement for the management of DKA should be modified according to the closely monitored fluid status of patients with COVID-19.

#### 3.2.3. Pharmacological Therapy for DM

**Figure 4:**
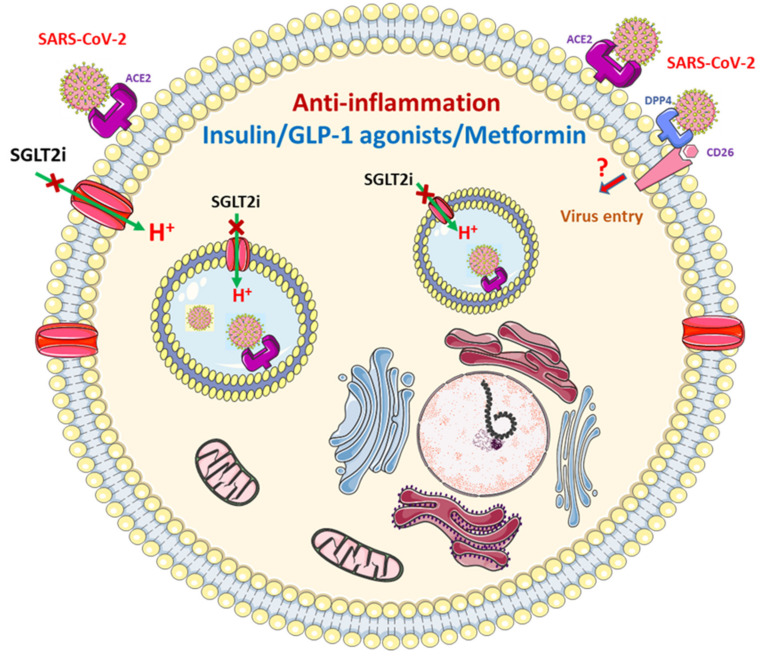
Possible beneficial mechanisms of diabetic medicines on host cells in patients with COVID-19.

The possible beneficial mechanisms of oral hypoglycemic agents on host cells in patients with COVID-19 was demonstrated in Figure 4. Insulin has been extensively used to control glucose in hospitalized patients with infections. Insulin receptor signaling is critical for the functional potential of T cells during inflammation and acute infection [62]. Intensive insulin therapy has significant anti-inflammatory effects during critical illness [63]; hence, it should not be discontinued in patients with DM who develop COVID-19 and should be considered for patients receiving oral antidiabetic drugs who have a poor glycemic profile. The dose of insulin should be carefully adjusted based on regular monitoring of blood glucose to reach therapeutic goals.

Glucagon-like peptide-1 (GLP-1) receptor agonists reduce local or systemic inflammation in patients with type 2 DM or obesity [64]. However, GLP-1 plasma levels were elevated in critically ill patients with sepsis, which independently predicted the outcome of these patients [65]. The therapeutic effect of GLP-1 receptor agonists to reduce food intake and promote weight loss [66] may exert a negative effect on patients with severe COVID-19. Therefore, GLP-1 receptor agonists should be used cautiously in patients with COVID-19 and it should be ensured that such patients have adequate fluid intake and regular meals.

Metformin exerts anti-inflammatory effects irrespective of DM status [67]. However, anorexia, dehydration and rapid deterioration in clinical status secondary to SARS-CoV-2 infection pose a risk of lactic acidosis in patients receiving metformin therapy. Thus, the fluid status and renal function of patients with DM and COVID-19 who receive metformin therapy must be carefully monitored. Metformin should be discontinued in patients with severe COVID-19 or an unstable hemodynamic condition.

Sulfonylureas stimulate insulin secretion from pancreatic β-cells. However, increased insulin resistance due to acute inflammation or infection may reduce the therapeutic effect of sulfonylureas. Patients with COVID-19 who have symptoms of anorexia may have an increased risk of hypoglycemia related to sulfonylureas. Hence, sulfonylureas should be avoided for patients with DM who have severe COVID-19.

## 4. Role of Dipeptidyl Peptidase-4 (DPP4) Inhibition

DPP4, namely cluster of differentiation 26 (CD26), a cell-surface protease belonging to the prolyl oligopeptidase family, cleaves dipeptides from the N-terminus [68]. A recent study demonstrated that SARS-CoV-2 enters and hijacks the target cell through a possible tight interaction between the N-terminal S1 domain of S protein and the DPP4/CD26 surface [69]. Another study reported the potential use of DPP4 as a candidate binding site for SARS-CoV-2 in the course of virus entry [70]. Several benefits of DPP4 inhibitors for patients with DM who develop COVID-19 have been proposed, including reduction of cytokine overproduction, downregulation of macrophage activity, enhancement of GLP-1 anti-inflammatory activity and stimulation of pulmonary anti-inflammatory effects [71]. A study demonstrated that infection of human primary bronchiolar epithelial cells by human coronavirus, at the Erasmus Medical Center, was inhibited by DPP4 antibodies in a dose-dependent manner [72]. However, no real-world data have confirmed the effect of DPP4 inhibitors on COVID-19 infection outcomes. Further studies regarding the effect and prognosis of DPP4 inhibitors in patients with DM and COVID-19 are warranted.

## 5. COVID-19 and SGLT2 Inhibitor

SGLT2i reduced renal glucose reabsorption and plasma glucose. The increased glucagon/insulin ratio secondary to SGLT2i use [73] and the reduced renal excretion of ketone bodies [74] contributed to the increased risk of DKA in SLT2i users, compared with non-users [75]. A previous report stated that patients with DM receiving SGLT2i who had symptoms of infection developed DKA [76]. However, the potential benefits of SGLT2i, including amelioration of inflammation, oxidative stress and hypoxia of tissue through downregulation of adipokines and cytokines have been proposed [77]. Other potential beneficial effects of SGLT2i for patients with COVID-19 who have interstitial pulmonary edema, such as increased hematocrit and selective reduction of interstitial volume without significant changes in blood volume, have also been reported [78]. The potential protective effect of an SGLT2i, dapagliflozin, through reduction in lactate level and prevention of lowering of cytosolic pH may be mediated by several mechanisms, such as the reduction of oxygen consumption in tissues, the promotion of the aerobic metabolism of glucose, the reduction of lactate production, an increase in urinary lactate excretion and a reduction of activation of lactate/H^+^ symporter [79]. A previous study summarized the rationale for using SGLT2i for patients with DM who develop COVID-19; it reduces serum lactate levels, inhibits Na^+^/H^+^ exchanger activity, activates the alternative renin–angiotensin–aldosterone system pathway by activating ACE2 and reduces proinflammatory cytokine levels [80]. Based on current knowledge about the benefits and pitfalls of SGLT2i for patients with DM who develop COVID-19, SGLT2i treatment should be continued or introduced to achieve adequate glucose control in patients with DM with mild or asymptomatic COVID-19 and it should be discontinued in patients with more severe COVID-19 who have symptoms or signs of decreased appetite, dehydration, hypoxia, sepsis and impaired renal function.

## 6. Practical Recommendations

Patients with DM who develop COVID-19 should be divided into those with mild or asymptomatic COVID-19 and those with more severe COVID-19 who show symptoms or signs of decreased appetite, dehydration, hypoxia, sepsis and impaired renal function during either inpatient or outpatient treatment. For patients with mild or asymptomatic COVID-19, single or combination therapy with metformin, SGLT2i, and DDP4i should be considered to achieve favorable glycemic control and to take advantage of the other possible beneficial effects of these drugs. Clinicians should be cautious about the use of metformin and SGLT2i in patients with DM who have more severe COVID-19; intensive monitoring of glucose, fluid status, and renal function of these patients is warranted. Insulin-based treatment is recommended and cessation of metformin and SGLT2i should be considered for patients with DM and COVID-19 who require hospitalization to avoid the potential adverse effects of these drugs. As limited information is available regarding the outcome of patients with DM who develop COVID-19 and receive a specific category of medication for DM, further evidence from randomized or case–control (clinical) trials is necessary to elucidate the effectiveness and pitfalls of the different types of medication for DM.

## Abbreviations

A disintegrin and metalloproteinase 17	ADAM17
Angiotensin 1–7	Ang 1–7
Angiotensin-converting enzyme 2	ACE2
Cluster of differentiation 26	CD26
Coronavirus disease 2019	COVID-19
Diabetes mellitus	DM
Diabetic ketoacidosis	DKA
Dipeptidyl peptidase-4	DPP4
Glucagon-like peptide-1	GLP-1
Hexosamine biosynthetic pathway	HBP
Interferon regulatory factor–5	IRF5
Middle East respiratory syndrome	MERS
Renin–angiotensin system	RAS
Severe acute respiratory syndrome	SARS
Severe acute respiratory syndrome coronavirus 2	SARS-CoV-2
Sodium–glucose cotransporter 2 inhibitor	SGLT2i
Spike	S
Transmembrane serine protease 2	TMPRSS2
